# Effects of Nb on the Microstructure and Compressive Properties of an As-Cast Ni_44_Ti_44_Nb_12_ Eutectic Alloy

**DOI:** 10.3390/ma12244118

**Published:** 2019-12-09

**Authors:** Shifeng Liu, Song Han, Liqiang Wang, Jingbo Liu, Huiping Tang

**Affiliations:** 1 School of Metallurgical Engineering, Xi’an University of Architecture and Technology, No.13 Yanta Road, Xi’an 710055, China; Liushifeng66@xauat.edu.cn (S.L.); thpfys@126.com (H.T.); 2 State Key Laboratory of Metal Matrix Composites, School of Material Science and Engineering, Shanghai Jiao Tong University, No. 800 Dongchuan Road, Shanghai 200240, China

**Keywords:** NiTi–Nb, eutectic phase, superelastic, compressive properties

## Abstract

The addition of Nb can form a eutectic phase with a NiTi matrix in a NiTi-based shape memory alloy, improving the transition hysteresis of the NiTi alloy. A Ni_44_Ti_44_Nb_12_ ingot was prepared using the vacuum induction melting technique. Under compression deformation, the yield strength of the NiTi–Nb alloy is about 1000 MPa, the maximum compressive strength and strain can reach 3155 MPa and 43%, respectively. Ni_44_Ti_44_Nb_12_ exhibited a superelastic recovery similar to that of the as-cast NiTi_50_. Meanwhile, the loading–unloading cycle compression shows that the superelastic recovery strain reached a maximum value (2.32%) when the total strain was about 15%, and the superelasticity tends to rise first and then decrease as the strain increases.

## 1. Introduction

**Figure 1:**
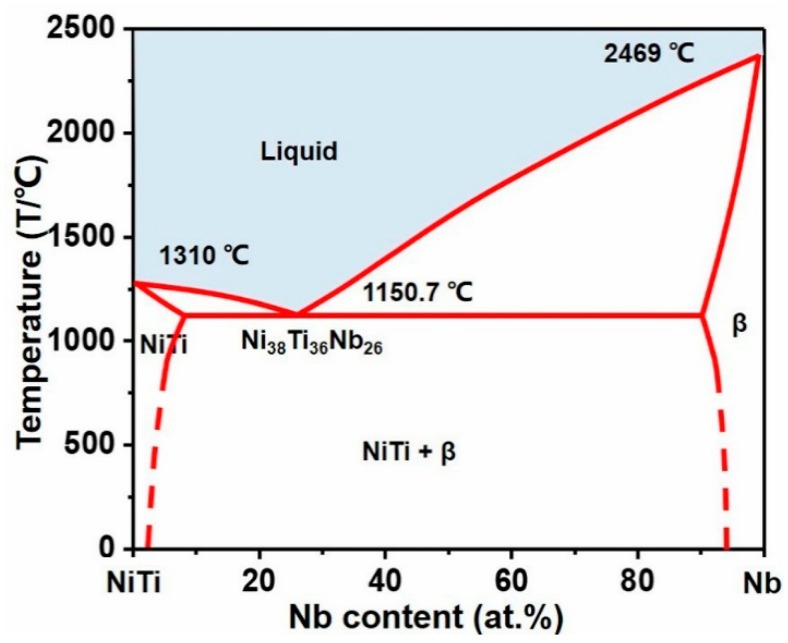
Pseudobinary phase diagram of the NiTi–Nb system [18] (reproduced with permission from ref. [18] of Elsevier).

NiTi alloys represent an important type of shape memory alloys (SMA), with unique superelasticity and a distinct shape memory effect. They have attracted considerable attention in aerospace engineering and the industrial and biomedical fields [1,2,3,4,5]. Compared with other SMAs, such as iron-based and copper-based alloys, NiTi-based SMA have high strength and plasticity, good corrosion resistance, and excellent biocompatibility. Therefore, they have a great potential for biomedical applications [6,7,8,9]. As in typical NiTi-based ternary alloys, Nb plays an important role in NiTi–Nb alloys, because the addition of Nb increases the transition hysteresis of NiTi alloys [10,11]. Moreover, the superior biocompatibility of niobium is conducive to the further improvement of biocompatibility in NiTi-based alloys [12,13,14,15,16,17]. Therefore, there is a strong motivation to develop NiTi–Nb ternary eutectic alloys. Based on the NiTi–Nb pseudobinary eutectic phase diagram in Figure 1, the NiTi–Nb eutectic phase was formed by Nb and NiTi at a high temperature. The phase diagram shows that the eutectic temperature is far lower than the melting point of Ni, Ti, and Nb, and the reaction temperature can reach 1180 °C to ensure the formation of the eutectic phase [18,19].

The current research has focused extensively on the work of classical alloys such as Ni_47_Ti_44_Nb_9_ and Ni_x_Ti_y_Nb_z_ (z < 5%) [11,20,21,22]. Fan et al. [21] found that different Ni/Ti ratios (x/y = 1, 1.0210, 1.0425, 1.0645, and 1.0869) in NiTi–Nb alloys lead to the formation of different eutectic regions and that β-Nb (the main form of Nb-rich phase, with a body-centered cubic structure) precipitates in the NiTi matrix. Extra Ni tends to form more Ni-rich precipitates, such as Ni_4_Ti_3_, Ni_3_Ti_2,_ and Ni_3_Ti. Furthermore, the formation of a Ni-rich precipitate and the evaporation of Ni lead to high Ti content in the NiTi matrix [22]. Zhang et al. [22] studied the effects of the Nb content (1, 3, 5 atom %) on the precipitation and hardening behavior of the Ni-rich Ni_55_Ti_45_ (atom %) alloy and found that a large number of nanoscale Ni_4_Ti_3_ precipitates are the main factors causing the high hardness of these alloys. In addition, Wang et al. [23] studied the microstructure evolution and superelastic properties of porous NiTi–Nb and found that a large number of dislocations and stacking faults were formed between the Nb-rich phase and the eutectic phase, which accelerated the eutectic phase transition. The mechanical properties of the NiTi–Nb eutectic alloy under monotonic compression were obtained by Bewerse et al. [24], and the yield strength was 630 MPa, while the stress was 1080 MPa when the applied strain was 14.7%.

However, the increase of Nb can significantly increase the formation of a NiTi–Nb eutectic phase, which can significantly improve martensite transformation [18,23]. Accordingly, 10 atom % Nb can be dissolved into the NiTi matrix at the eutectic temperature, and excessive Nb addition leads to more β-Nb phase separation [18,25]. Therefore, an interesting and rarely reported strategy consists in regulating the microstructure and mechanical properties of the NiTi alloy by adding 10% or more of Nb content. In this work, a cast ingot was prepared with nominal composition Ni_44_Ti_44_Nb_12_ (atom %, the same hereafter) in a vacuum induction furnace. The microstructure and phase composition of the alloy were analyzed with an optical microscope and a scanning electron microscope. Vickers microhardness and uniaxial compression were used to study the mechanical properties and superelastic recovery.

## 2. Experimental Details

A nominal composition of the Ni_44_Ti_44_Nb_12_ (atom %) eutectic alloy was prepared using the vacuum induction melting technique. The vacuum induction melting temperature reached 1200 °C, and the stirring was repeated several times to ensure the uniformity of the composition. After being ground and polished, the Ni_44_Ti_44_Nb_12_ eutectic alloy sample was etched by a reagent (~10% HNO_3_, 20% HF, and 70% H_2_O) for ~15 s. The etched sections were imaged using an optical microscope (Nikon MA200 Eclipse, Tokyo, Japan) and scanning electron microscopes (SEM, Hitachi 8030, Tokyo, Japan). Meanwhile, the phase composition was obtained by X-ray diffraction (XRD) using a Regaku D/max 2200 pc diffractometer (Tokyo, Japan). The XRD radiation source was Cu–Kα, and the angle range was 30–80°.

The Vickers microhardness tests were carried out by a XHVT-1000Z intelligent microhardness device (ShangCai, Shanghai, China) at a load of 1 kg for 15 s. The mechanical properties of the samples were assessed through uniaxial compressive tests on an MTS servo-hydraulic machine (Eden Prairie, MN, USA). Furthermore, cyclic loading–unloading compression was carried out on the NiTi–Nb sample, whose dimension was Φ4 × 6 mm. The cyclic compressive strains during loading–unloading testing were 3%, 6%, 9%, 12%, 15%, 18%, and 21%, respectively. All the samples were compressed at a loading rate of 0.1 mm/min at room temperature.

## 3. Results and Discussion

### 3.1. Microstructure of the As-Cast Ni_44_Ti_44_Nb_12_ Alloy

**Figure 2:**
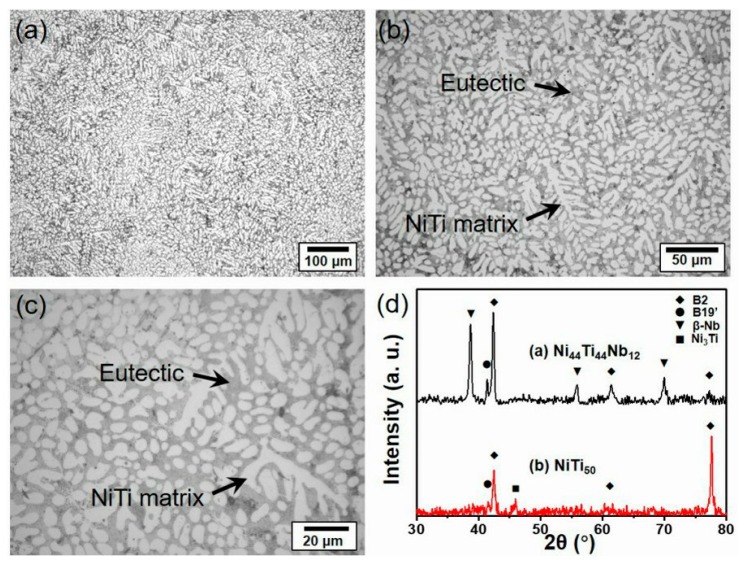
The microstructure of the as-cast NiTi–Nb alloy under an optical microscope: (**a**) 200×, (**b**) 500×, (**c**) 1000×, and (**d**) XRD patterns of the as-cast Ni_44_Ti_44_Nb_12_ and the as-cast NiTi_50_.

Figure 2 shows the microstructure of the as-cast Ni_44_Ti_44_Nb_12_ alloy under an optical microscope. It can be found that a large number of white rod-shaped NiTi matrix phases exist in the Ni_44_Ti_44_Nb_12_ sample (Figure 2a). Around the NiTi phase, there is a gray eutectic phase. As shown in Figure 2b,c, the rod-like and dendritic NiTi matrix phases can be more clearly observed. In the study of Ni_50−x/2_Ti_50−x/2_Nb_x_ (x = 0, 5%, 10%, 15%, 20%, and 25%, atom %), with the increase of Nb content, the NiTi phase gradually decreased, but the gray eutectic region gradually increased, accompanied by the gradual refinement of the grain size of NiTi. The rod-like NiTi phase began to appear when the Nb content reached 10% (atom %) [18]. In the fluctuation of 10%–15% Nb content, the NiTi phase was further reduced, and the grain size was also refined from ~30 to ~5 μm [18]. It can be seen that the results of the Ni_44_Ti_44_Nb_12_ component are consistent with previous work and further supplemented the detailed study. In addition, XRD detection was performed on Ni_44_Ti_44_Nb_12_ and NiTi_50_, and the results are shown in Figure 2. The diffraction pattern of NiTi_50_ has five peaks at 42°, 42.4°, 45.9°, 61.2°, and 77.6°, respectively. The peaks of 42.4°, 61.2°, and 77.6° are well indexed to the B2 phase and the peaks at 42° and 45.9° to the B19′ phase and Ni_3_Ti. It can be seen that the NiTi_50_ sample is mainly a B2 phase with a small amount of B19′ phase and a Ni_3_Ti precipitate phase, respectively. In the result of the Ni_44_Ti_44_Nb_12_ sample, the peaks appearing at 37.8°, 55.2°, and 69.9° can be clearly indexed to the β-Nb phase. Compared with NiTi_50_, the Ni_44_Ti_44_Nb_12_ sample is mainly composed of B2 phase and β-Nb phase with a small amount of B19′ phase, but no brittle precipitation such as Ni_3_Ti and Ni_4_Ti_3_ was detected.

**Figure 3:**
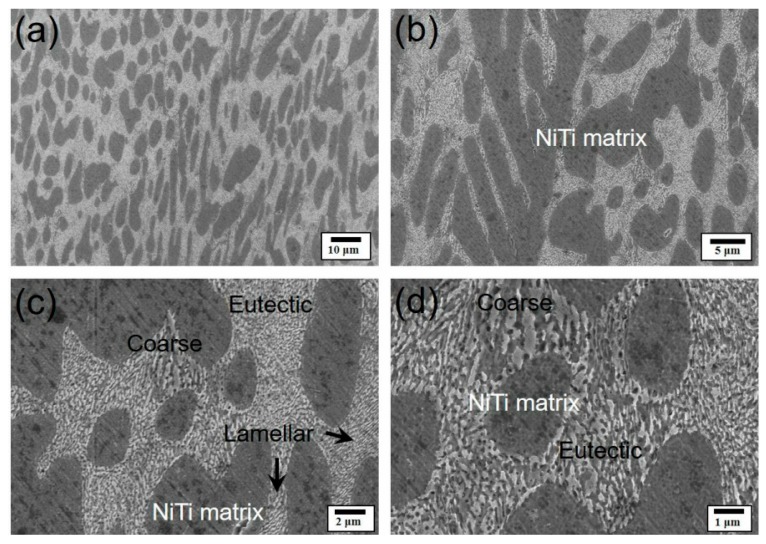
SEM microstructure of the as-cast NiTi–Nb alloy: (**a**) 1000×, (**b**) 2000×, (**c**) 5000×, and (**d**) 10,000×.

Figure 3 shows the SEM microstructure of the as-cast NiTi–Nb alloy. The eutectic morphology is more clearly shown in Figure 3a–d. In Figure 3a, the matrix phase mainly exists as a rod-like shape, the rest being a bright NiTi–Nb eutectic structure. Figure 3b clearly shows the gray dendritic matrix phase, with a large number of eutectic phases distributed around it. The different magnified graphs of the eutectic regions near the rod-like and dendritic NiTi matrix are shown in Figure 3c,d, respectively. In addition, a coarser microscopic phase is found at the boundary of the eutectic region, composed of Nb-rich precipitates. These equiaxed phases have a diameter of about 1 μm. Meanwhile, some lamellar precipitate phases with a length of ~2 μm were found. In general, it can be seen that the eutectic phases are relatively uniform in the eutectic region. The shape is mainly granular and lamellar, which is consistent with the results reported in [23,26,27].

### 3.2. Mechanical Properties

#### 3.2.1. Vickers Microhardness

**Figure 4:**
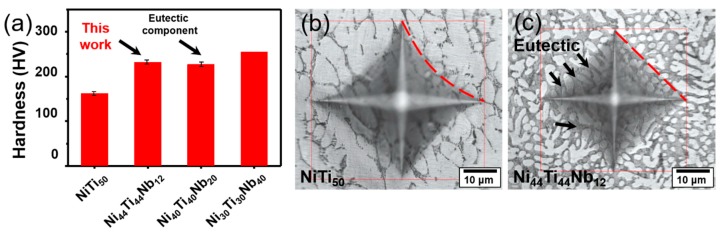
Vickers hardness and indentations: (**a**) the Vickers hardness of NiTi_50_, Ni_44_Ti_44_Nb_12_, Ni_40_Ti_40_Nb_20_ [24], and Ni_30_Ti_30_Nb_40_ [24], (**b**) the indentation of NiTi_50_, and (**c**) the indentation of Ni_44_Ti_44_Nb_12._

Figure 4 shows the Vickers hardness and indentations of different samples. The Vickers hardness values measured on the polished surfaces of NiTi_50_ and Ni_44_Ti_44_Nb_12_ were 162 ± 6 and 232 ± 7 HV (Indentation surface area divided by load value), respectively (Figure 4a). Comparing the hardness of the eutectic components (227 ± 8 HV) and that of the cast Ni_30_Ti_30_Nb_40_ (255 HV) [24], the hardness of Ni_44_Ti_44_Nb_12_ was within a reasonable range. The hardness was averaged over 20 indentations, with a span of more than 60 μm across multiple eutectic phases and the NiTi substrate. The hardness was therefore a measurement of the mixture of the eutectic phase (indicated by the black arrows) and the NiTi matrix phase. In the hardness test, the samples left a clear indentation when the indenter penetrated the material, and the surface of the material was deformed around the indentation [28,29]. The indentation edge of NiTi_50_ is significantly more concave than that of Ni_44_Ti_44_Nb_12_, which is shown by the red line. The more severe indentation deformation of NiTi_50_ confirms that its hardness is lower than that of Ni_44_Ti_44_Nb_12_. In a study on the hardness of NiTi in various compositions, it was found that NiTi maintains a B19′ martensite hardness of 234 HV at room temperature, while the B2 austenite state is 186 HV at room temperature [30]. It is believed that the as-cast NiTi_50_ is austenized at room temperature, which is consistent with the XRD results of NiTi_50_ (Figure 2d). Meanwhile, it was found that the hardness of Ni_44_Ti_44_Nb_12_ (232 ± 7 HV) was slightly higher than those of the as-cast NiTi_50_ and the NiTi–Nb eutectic composition, but lower than that of the Ni_30_Ti_30_Nb_40_ cast sample.

#### 3.2.2. Compressive Properties

**Figure 5:**
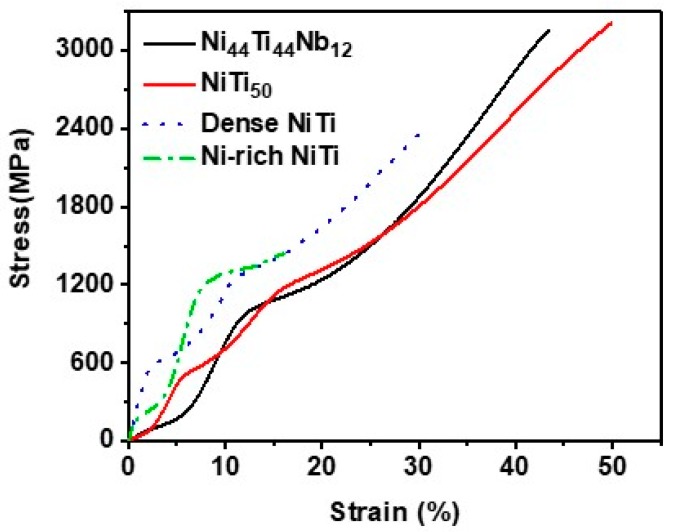
Uniaxial compression stress–strain curve of the Ni_44_Ti_44_Nb_12_ samples compared with compression stress–strain curves for as-built NiTi_50_, dense NiTi [7], and Ni-rich NiTi [28].

Figure 5 shows the Ni_44_Ti_44_Nb_12_ stress–strain curves of the NiTi_50_ and Ni_44_Ti_44_Nb_12_ samples. The corresponding values of yield stress (σ_0.2_), ultimate compressive stress (UCS), and engineering strain (ES%) were 960 MPa, 3155 MPa, and 43%, respectively. The initial load was nonlinear and its slope increased up to about 200 MPa. The stress–strain curve of Ni_44_Ti_44_Nb_12_ shows a significant platform region after initial deformation at 200–300 MPa, as well as a yield region when the stress reached 960 MPa. According to the literature [31,32], the stress–strain platform (Figure 5) is caused by the stress-induced NiTi alloy’s martensitic transformation. A martensitic strain platform with lower strength appeared in the Ni_44_Ti_44_Nb_12_ sample, indicating that the addition of Nb required a smaller phase change driving force for martensitic transformation. With the increase of the Nb content (0%–15%), the martensitic transformation’s start temperature gradually decreased [21]. The replacement of Ni atoms by Nb atoms in the solid solution phase of NiTi resulted in the release of Ni and increased the Ni/Ti ratio [21]. The Ni/Ti ratio could result in lowering the martensitic transformation temperature, so the addition of Nb reduces the driving force of the martensite phase transformation. Moreover, Figure 5 shows stress–strain curves for the as-cast NiTi_50_, dense NiTi [7], and Ni-rich NiTi [28]. Our work on as-cast NiTi_50_ showed a compression curve similar to that of Ni_44_Ti_44_Nb_12_, while the dense NiTi stress–strain curve also showed a yield strength and curve characteristic that are substantially consistent with NiTi_50_. Furthermore, Ni_44_Ti_44_Nb_12_ can exhibit a higher yield strength with a lower compressive strain. It was found that the strength of the alloy first decreases and then increases as the Nb content increases. This seems to be caused by the dissolution of Nb in the NiTi matrix to form a eutectic phase, which regulates the phase structure [23]. However, considering the balance between material strength and toughness, Ni_44_Ti_44_Nb_12_ is a component that can be further explored.

**Figure 6:**
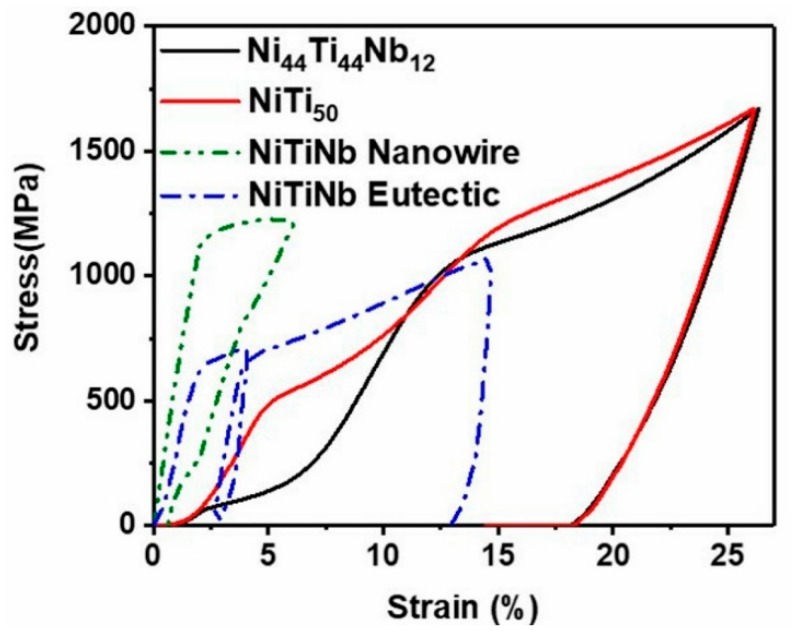
Uniaxial loading–unloading compression stress–strain curve, compared with compression stress–strain curves for NiTi–Nb eutectic [24] and NiTi–Nb nanowire composite [33].

The stress–strain curves for as-cast Ni_44_Ti_44_Nb_12_, as-cast NiTi_50_, NiTi–Nb eutectic [24], and highly textured NiTi–Nb eutectic nanowires [33] are shown in Figure 6. The Ni_44_Ti_44_Nb_12_ sample exhibited a stress platform at a compressive stress of 200 MPa, which is consistent with the result in Figure 5, indicating the beginning of the martensitic transformation. According to the stress–strain curve of the Ni_44_Ti_44_Nb_12_ sample (Figure 6), when the deformation strain reached about 6%, the austenite was almost completely transformed into martensite. Then, the stress increased linearly with the strain. Subsequently, the Ni_44_Ti_44_Nb_12_ strain was 26.3%, and the superelastic recovery began. After unloading, the residual plastic strain was 18%. In the highly textured NiTi–Nb eutectic nanowires, the alloy yield strength was 1140 MPa. It has been found that the addition of nanomaterials has a significant strength enhancement effect on the material. However, in the compression recovery process, the expected superelastic response was not found. Under two consecutive loading–unloading compressions of the NiTi–Nb eutectic composition, the alloy exhibited slight elastic recovery and superelastic recovery after yielding at 630 MPa [24]. The stress platform of the Ni_44_Ti_44_Nb_12_ was different from those of the NiTi–Nb eutectic [24] and the highly textured NiTi–Nb nanowires [33], but the superelastic recovery of this alloy was similar to that of NiTi_50_. Therefore, the addition of the appropriate amount of Nb element did not have a severe adverse effect on the superelastic recovery of the NiTi alloy while reducing the martensitic strain driving force.

**Figure 7:**
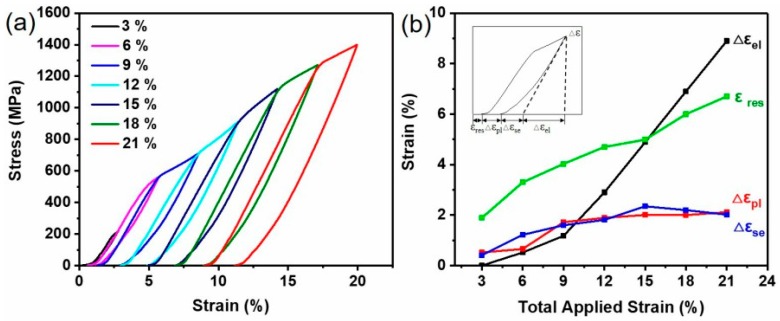
(**a**) Cyclic compression loading–unloading stress–strain curve of the NiTi–Nb eutectic alloy, (**b**) comparison of strain after unloading. ε_res_: the accumulated residual plastic strain of Ni_44_Ti_44_Nb_12_; Δε_pl_: the residual plastic strain; Δε_el_: the elastic recovery strain; and Δε_res_: the superelastic recovery strain. The inset is a schematic representation of the individual strain components.

Figure 7a shows the stress–strain curves of the as-cast Ni_44_Ti_44_-Nb_12_ after successive loading–unloading. The cyclic compressive strains during loading–unloading testing are 3%, 6%, 9%, 12%, 15%, 18%, and 21%, respectively. In each successive loading–unloading cycle, residual plastic strain began to accumulate during the previous cycle. High superelastic recovery and elastic recovery were due to more martensites appearing during deformation, while the smaller residual strain during unloading showed that the de-twinning of martensite was close to full recovery [23]. The Ni_44_Ti_44_Nb_12_ sample was strengthened by plasticity for the subsequent cycles, and the cumulative residual strain indicates that the Ni_44_Ti_44_Nb_12_ sample had irreversible plastic deformation [23]. Figure 7b shows the strain of the superelastic recovery and the elastic recovery after unloading. It can be clearly found that when the strain was 21%, ε_res_ and Δε_el_ reached a maximum value of 6.7% and 8.9%, respectively. When the total applied strain reached 9%, Δε_pl_ rose slowly and eventually to 2.1 %. The superelastic recovery strain reached its maximum when the total strain was about 15%, showing a trend of first rising and then falling as the strain increased.

## 4. Conclusions

In this work, the nominal composition of the Ni_44_Ti_44_Nb_12_ (atom %) eutectic alloy was prepared by vacuum induction in a melting furnace, based on the NiTi–Nb pseudobinary eutectic phase diagram. The main results, obtained through the analysis of the phase transformation and deformation mechanisms, are summarized as follows:

1. The Ni_44_Ti_44_Nb_12_ eutectic alloy has a NiTi phase and a uniform eutectic phase. As a result of the Vickers microhardness of the sample, the addition of Nb increased Ni_44_Ti_44_Nb_12_ by about 70 HV relative to NiTi_50_. Comparing the higher Nb content of Ni_40_Ti_40_Nb_20_ and Ni_30_Ti_30_Nb_40_, it was found that the hardness of Ni_44_Ti_44_Nb_12_ (232 ± 7 HV) was slightly higher than that of the NiTi–Nb eutectic composition (Ni_40_Ti_40_Nb_20_) and lower than that of the Ni_30_Ti_30_Nb_40_ casting sample. The hardness of A was kept within a reasonable range.
2. In uniaxial compression, the NiTi–Nb alloy had a yield strength of about 1000 MPa and a maximum compressive strength and strain of 3155 MPa and 43%, respectively. The martensitic transformation platform of Ni_44_Ti_44_Nb_12_ at 200 MPa indicates that the composition of NiTi–Nb alloy required a smaller martensitic transformation driving force, while the superelastic strain recovery similar to that of the as-cast NiTi_50_ indicates that the addition of the appropriate amount of Nb did not have a severe adverse effect on the superelastic recovery.
3. The loading–unloading cycle compression showed that the superelastic recovery strain reached a maximum value (2.32%) when the total strain was about 15% and that the superelasticity tends to rise first and then decrease as the strain increases.
